# Nasal breathing: a neglected factor in metabolic regulation?

**DOI:** 10.1007/s00405-024-09093-y

**Published:** 2024-12-02

**Authors:** Francisco Alves de Sousa, João Tavares Correia, Miguel Gonçalves Ferreira, Marta Rios, Manuel Magalhães, Mariline Santos

**Affiliations:** 1 Otorhinolaryngology and Head & Neck Surgery, Centro Hospitalar Universitário de Santo António, Largo do Prof. Abel Salazar, 4099-001 Porto, Portugal; 2 Head of Sleep Medicine Laboratory, Pediatrics Department of Centro Materno Infantil do Norte, Unidade Local de Saúde de Santo António, Largo do Prof. Abel Salazar, 4099-001 Porto, Portugal; 3 Pneumology Unit and Neonatology Unit, Paediatrics Department, Centro Materno Infantil do Norte (CMIN), Centro Hospitalar Universitário de Santo António, Largo da Maternidade de Júlio Dinis 45, 4050-651 Porto, Portugal

**Keywords:** Nasal breathing, Resting metabolic rate, Septoplasty, Turbinate reduction, Rhinoplasty, Peak nasal inspiratory flow

## Abstract

**Purpose:**

Nasal breathing (NB) is a fundamental physiological process, and emerging research indicates its potential role in modulating resting metabolism, impacting energy expenditure and metabolic efficiency. This study investigates the impact of NB on resting metabolic rate (RMR), offering novel insights into metabolic regulation.

**Methods:**

A prospective study was conducted on patients undergoing nasal surgery, with measurements taken before and 3 months after surgery. Metabolic rate assessments, anthropometric dimensions, and peak nasal inspiratory flow (PNIF) were recorded. Factors like age, sex, and health status were considered to control for confounding variables.

**Results:**

A total of 83 patients were initially enrolled: 17 underwent septorhinoplasty (SRP), 61 septoplasty (ST) and 5 inferior turbinate reduction alone. 72 patients completed the follow-up. SRP patients exhibited significantly higher pre- and post-operative RMR compared to ST patients (p = 0.005), and this association was not observed when PNIF was included in the analysis (p > 0.05). Pre-operative and post-operative PNIF values significantly correlated with pre-operative and post-operative RMR (p = 0.049 and p = 0.005, respectively). Post-operative PNIF predicted post-operative RMR after confoundment adjustment in linear regression (β = – 0.043, p = 0.017). Importantly, total body weight increased after surgery (pre-op: 74 ± 14.6 kg versus post-op: 75.6 ± 15.5 kg, p < 0.001) due to an increment in muscle mass (pre-op: 52.3 ± 12 versus post-op: 55.5 ± 14, p < 0.01).

**Conclusion:**

Preliminary analysis suggests a potential link between NB and RMR, emphasizing the overlooked role of nasal respiratory physiology in energy homeostasis. Surgery also elicited body composition alterations. Further research is needed to uncover the underlying mechanisms of this association. Understanding the impact of NB on RMR could underscore its significance in metabolic regulation, reinforcing the importance of nasal surgery on overall health. This study provides foundation for future investigations.

## Introduction

Nasal breathing (NB) offers a multitude of well-established benefits, from optimizing air quality through filtration, humidification, and warming [1, 2] to facilitating efficient pulmonary oxygenation via nitric oxide production (NO) [3]. Recent research has extended beyond these fundamental functions to explore the potential impact of NB on cardiopulmonary metabolism [4–8], with studies demonstrating its influence on cardiovascular parameters such as blood pressure, heart rate variability [9], and both right [7] and left [8] ventricular myocardial performance.

While the connection between NB and cardiovascular function is increasingly recognized, its influence on metabolic processes remains largely unexplored. The nasal passages play an active role in optimizing lung gas exchange [10]. NB has been associated with increased NO levels, a molecule that promotes broncho and vasodilation and enhances blood flow, facilitating oxygen exchange [3, 11, 12]. NB also influences neural pathways, including the trigeminal-vagal reflex, which can impact cardiovascular function [13]. The precise impact of nasal obstruction on this reflex remains to be elucidated. These observations suggest that NB might impact cardiovascular efficiency and contribute to metabolic regulation.

This study focuses on the impact of NB on resting metabolic rate (RMR), as exploration of the direct effects of NB on metabolism remain very limited. Could nasal breathing be associated with resting metabolic rate (RMR)? Could it affect the utilization of different energy substrates? The primary aim is to provide insights into the so far unexplored relationship between nasal breathing and resting metabolic processes by two main methods: (1) measure NB and RMR to assess a potential association; (2) evaluate the effect of surgery on RMR and body composition.

## Material and methods

### Study design and participants

This prospective observational study enrolled adult patients experiencing nasal obstruction due to septal deviation and/or inferior turbinate hypertrophy who did not respond to medical management with nasal steroids. Data was collected from January to December 2023. Participants were selected based on availability and met specific inclusion criteria. To minimize the potential confounding effects of other nasal pathologies on metabolic function, patients with a history of chronic rhinosinusitis or any other nasal condition requiring additional surgical intervention (e.g., functional endoscopic sinus surgery) were excluded. Patients with a history of previous nasal surgery were also excluded to avoid potential confounding effects from prior surgical interventions. Patients with autoimmune diseases and/or using chronic systemic corticosteroid or biologic treatments were also excluded from the study. Other exclusion criteria included associated comorbidities (craniofacial or thoracic dysmorphisms, cognitive or neurological impairments).

The study population included individuals undergoing three types of nasal surgery: inferior turbinate reduction (ITR) alone, septoplasty (ST) combined with ITR, and septorhinoplasty (SRP) combined with ITR.

### Surgical technique and post-operative care

**Inferior Turbinate reduction Group (ITR):** Inferior turbinate reduction was performed using radiofrequency plasma ablation (BONSS® system) without septal intervention.

**Septoplasty Group (ST):** Septoplasty was performed using the Cottle technique, with inferior turbinate reduction via radiofrequency plasma ablation (BONSS® system).

**Septorhinoplasty Group (STR):** Septoplasty was performed using the Cottle technique, combined with rhinoplasty utilizing the "spare roof technique—B" developed by Gonçalves Ferreira et al. [14]. ITR was carried out via monopolar electrocoagulation.

Internal silicone splints (Silastic®) were employed to stabilize the corrected septal position (in the septoplasty and septorhinoplasty groups) and mitigate postoperative complications such as bleeding and hematoma formation. Nasal packing (PosiSep®X) was used for immediate hemostasis. Patients received instructions for nasal saline irrigation four times a day. The removal of splints and aspiration of packing were performed seven days post-surgery.

### Data collection

Both subjective and objective parameters were evaluated.

### Objective measurements

**Resting Metabolic Rate (RMR):** Indirect calorimetry was employed to measure RMR. A COSMED Fitmate PRO ™ device (COSMED – The metabolic company, Rome, Italy) was used. This is a validated method for RMR estimation [15]. The FitMate PRO® metabolic analyzer determines energy expenditure using a fixed respiratory quotient (VCO_2_/VO_2_) of 0.85 and the simplified Weir equation [16, 17]. The equipment performed a self-calibration procedure before each analysis. To ensure accurate measurement of resting metabolic rate, participants' faces were fitted with silicone masks covering both the mouth and nose. Participants were instructed to breathe normally during the assessment. The equipment included a respiratory rate sensor that allowed the investigator to monitor the participant's breathing pattern and initiate the recording once a steady respiratory state was achieved. To standardize conditions and minimize variability, participants were instructed to refrain from strenuous physical activity for 24 h prior to the assessment and to fast for at least 4 h before the measurement. RMR measurements were conducted in a quiet, temperature-controlled room with participants in a supine position. Following a 15-min acclimatization period, oxygen consumption (VO_2_) was measured for a period of 6 min. RMR, instantaneous values of Oxygen uptake (VO_2_), Ventilation (Ve), Respiratory, frequency (Rf) and fraction of O_2_ expired (FeO_2_) were recorded. To account for potential variations in body weight among participants, RMR values were normalized by dividing them by each individual's body weight in kilograms. This approach ensured that any observed differences in RMR were not simply a reflection of differences in body size after surgery, allowing for more accurate comparisons.

**Anthropometric Measurements:** Body composition was assessed using a handheld bioelectrical impedance analysis (BIA) device (Tanita UM–076, TANITA Corporation; Tokyo, Japan). Proper heel alignment with the electrodes was verified, and individual height, gender, and age were entered before each measurement. The foot-to-foot BIA method required participants to stand upright and barefoot on the platform containing the electrodes. Height was registered based on the national identity card information. The following parameters were obtained from the BIA measurements: weight (kilograms [Kg]), body fat percentage (%), total body water percentage (%), muscle mass (Kg), Harris-Benedict estimated resting metabolic rate (RMRpredicted) (Kcal), and visceral fat level. Body mass index (BMI) was also calculated.

**Peak Nasal Inspiratory Flow (PNIF):** PNIF was determined as the maximum airflow rate achieved through the nose during inspiration. An In-Check Nasal inspiratory flowmeter, a portable, FDA-approved device, was utilized for this purpose. This instrument quantifies the maximum airflow achievable through the nose during a forceful inhalation. Each In-Check meter undergoes individual calibration to ensure accuracy within ± 10% or 10 L/min (whichever is greater). The device's measurement range extends from 30 L/min to 370 L/min. Three measurements were taken, with the highest value recorded for analysis.

### Subjective measurements

**SNOT-22 Questionnaire:** The Sino-Nasal Outcome Test (SNOT-22) was used to assess patients' self-reported nasal and rhinologic symptoms and their impact on the quality of life [18].

**Epworth Sleepiness Scale (ESS):** The ESS was administered to evaluate patients' subjective level of daytime sleepiness [19].

**Short Form-36 (SF-36):** The SF-36 was utilized to capture a comprehensive picture of patients' health-related quality of life (HRQoL), encompassing eight domains like physical functioning, social functioning, and mental health [20].

Potential confounding factors such as age, sex, and comorbidities were also considered during the study. Primary outcome measurements were defined as nasal patency (measured by PNIF) and RMR-related measurements (obtained through indirect calorimetry). All the other variables were considered secondary outcome measurements.

### Timing and blinding of measurements

**Fig. 1:**
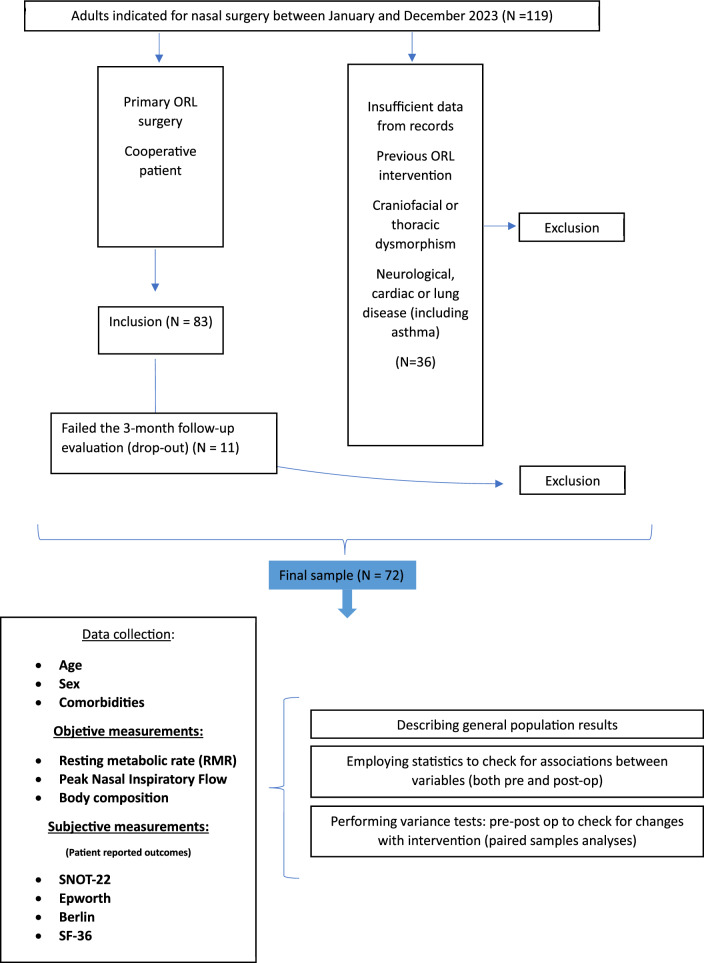
Methodological approach flow chart

The same operator performed all relevant measurements at two distinct time points: pre-operatively (on the day of the intervention) and post-operatively (3 months after surgery). During the collection of postoperative data, the researchers were blinded to the pre-operative results. Patients who did not attend the 3-month follow-up appointment were excluded from the final analysis (Fig. 1).

### Ethics

The study was approved by the Ethics Committee of CHUdSA/ICBAS (2021–234(186-DEFI/194-CE)) and adhered to the Declaration of Helsinki. Informed consent was obtained from all participants.

### Statistical analysis

Categorical data are shown as percentages, while continuous data are presented as means and standard deviations. Skewness and kurtosis were used to assess normality. Paired t-tests and Wilcoxon signed-rank tests were used to compare pre- and post-operative data. Change scores (∆) were calculated by subtracting postoperative scores from preoperative scores. Pearson´s or Spearman's correlation tests examined relationships between continuous variables. A t-test for paired samples was used to check the pre- to post-operative variance. Linear regressions were used as needed to account for potential confounders. Statistical analyses were performed using SPSS 29 (SPSS Inc.; Chicago, Illinois) for Windows, with statistical significance defined as p ≤ 0.05.

## Results

### Patient characteristics

A total of 83 patients were initially enrolled in the study: 17 underwent septorhinoplasty (SRP), 61 underwent septoplasty (ST), and 5 underwent inferior turbinate reduction (TR) alone. Seventy-two patients completed the 3-month follow-up assessment and were included in the final analysis (Fig. 1). Atopy was present in 12.5% of the sample, whereas PSG-confirmed OSA was present in only 2.8% of the sample. Other baseline characteristics of these 72 patients are summarized in Table 1.

**Table 1:** General description of the collected variables

Variable	
Female, n (%)	27 (37.5)
Age, years, mean (SD)	38.2 (14)
Height (cm)(SD)	172.6 (9.8)
Surgical group, n (%)	
Inferior turbinate reduction (ITR)	4 (5.6)
Septoplasty (ST)	54 (75)
Septorhinoplasty (STR)	14 (19.4)
Comorbidities, n (%)	
Atopy	9 (12.5)
Smoking	13 (18.1)
Diabetes Mellitus	3 (4.2)
Hypertension	7 (9.7)
Dislipidemia	2 (2.8)
Obesity	9 (12.5)
Obstructive sleep apnea	2 (2.8)
Thyroid disease	4 (5.6)
Chronic infectious disease	2 (2.8)

*SD* standard deviation

A comprehensive analysis of baseline comorbid conditions revealed no statistically significant differences between the surgical groups (p > 0.05 for all pathologies and inter-group comparisons).

### Pre-operative findings

#### Primary outcomes

The bivariate association test of pre-operative PNIF and RMR values showed a marginally significant negative correlation (r = – 0.218, p = 0.049). This association did not hold when potential confounders such as age, sex and height were included in a linear regression analysis (β = – 0.033, p = 0.149).

Pre-operatively, PNIF tended to be lower in the SRP group compared to the other groups (see Table 2), although this difference did not reach statistical significance (SRP vs ITR: p = 0.085; SRP vs ST: p = 0.109).

**Table 2:** Comparative analysis of pre- and post-operative values of outcome measurements

	Parameter	Pre-Op Mean (± SD)	Post-Op Mean (± SD)	P-Value
	(PNIF) (L/min)			
Nasal patency	Overall	70.8 ± 32.2	119.4 ± 44.3	< 0.001
	ITR	91.25 ± 33.8	135 ± 57.5	0.037
	ST	73.2 ± 32	123.9 ± 44	< 0.001
	STR	52.9 ± 27.1	93.8 ± 33.1	0.002
	Indirect calorimetry test			
	RMR (Kcal/Kg/day)			
Indirect calorimetry test	Overall	29.3 ± 6.8	29.6 ± 6.7	0.681
	ITR	26.6 ± 5.3	22.1 ± 4.4	0.119
	ST	28.4 ± 6.9	29.1 ± 6.4	0.396
	STR	34.1 ± 4.8	33.9 ± 5.8	0.593
	RMR/RMRpredicted (%)*			
	Overall	130.5 ± 25.9	133.4 ± 27	0.380
	ITR	128.5 ± 21.8	108.8 ± 19.3	0.142
	ST	126.1 ± 26.4	131 ± 26.6	0.231
	STR	148 ± 17	149.7 ± 22.7	0.759
	VO_2_ (ml/min)			
	Overall	307.5 ± 70	317.6 ± 73.6	0.205
	ITR	306 ± 71.1	268.8 ± 73.4	0.182
	ST	298.3 ± 69.4	312.4 ± 70.1	0.158
	STR	343.2 ± 65.1	351.7 ± 78.5	0.509
	Ve (L/min)			
	Overall	9.2 ± 2.5	9.5 ± 2.8	0.256
	ITR	10.1 ± 2.8	9.3 ± 4.1	0.674
	ST	8.8 ± 2.4	9.1 ± 2.7	0.475
	STR	10.2 ± 2.9	11.2 ± 2.4	0.066
	Rf (breaths/min)			
	Overall	14.3 ± 3.5	14 ± 4	0.612
	ITR	18.5 ± 3.2	19.2 ± 6.5	0.780
	ST	13.9 ± 3.6	13.7 ± 3.9	0.791
	STR	14.6 ± 2.1	13.7 ± 3.5	0.369
	FeO_2_ (%)			
	Overall	16.9 ± 0.7	16.9 ± 0.7	0.771
	ITR	17.3 ± 0.6	17.3 ± 0.7	0.942
	ST	16.9 ± 0.7	16.8 ± 0.7	0.409
	STR	16.8 ± 0.7	17.2 ± 0.4	0.049

*SD* Standard deviation, *PNIF* Peak nasal inspiratory flow, *RMR* resting metabolic rate, *VO*_*2*_ mean values of Oxygen uptake, *Ve* ventilation volume, *Rf* respiratory frequency, *FeO*_*2*_ fraction of O_2_ expired (FeO_2_)

*The values for RMR are compared to the predicted ones, calculated with the Harris-Benedict equation

p-value was a product of paired samples t-test

**Fig. 2:**
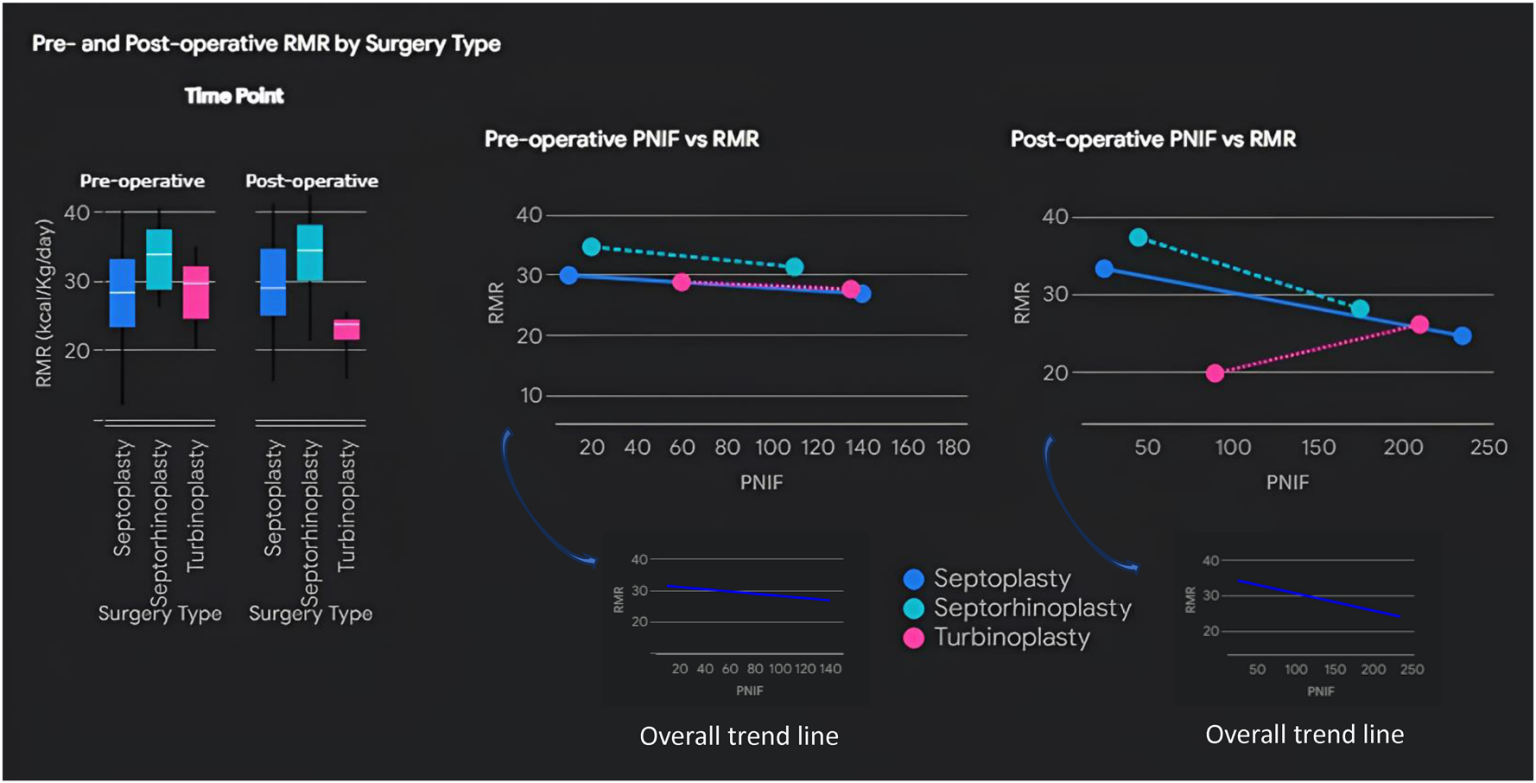
Pre- and post-operative Resting Metabolic Rate (RMR) and its relationship with Peak Nasal Inspiratory Flow (PNIF), stratified by surgery type. The colored dashed lines depict the linear regression trends for each surgery type, while the solid blue line represents the overall trend across all surgery types

Pre-operative RMR was significantly higher in the SRP group (34.1 ± 4.8 kcal/Kg/day) compared to the ST group (28.4 ± 6.9 kcal/Kg/day, p = 0.005). This significance persisted after adjusting for age, sex, and height (β = 3.879, p = 0.011). However, when pre-operative PNIF was included in the regression model, the association between SRP and higher pre-operative RMR was no longer significant (β = 2.920, p = 0.074). Figure 2 depicts RMR and PNIF relationship before and after surgery.

Resting metabolic rate (RMR) was higher in the SRT group (34.1 ± 4.8 kcal/Kg/day) than in the ITR group (26.6 ± 5.3 kcal/Kg/day), but this difference did not reach statistical significance (p = 0.103).

#### Secondary outcomes

No differences were found in any measurement of body composition (p > 0.05 for between groups) and baseline subjective measurements between groups (SNOT-22-IRT: 42 ± 10.2; ST: 45.2 ± 18.9, SRP: 31.3 ± 23, p > 0.05 for all comparisons; Epworth-IRT: 14 ± 3 vs ST: 9.6 ± 4.8, SRT: 7.3 ± 5, p > 0.05 for all comparisons; SF-36 – IRT: 23 ± 6; ST: 25 ± 4.1, SRP: 28 ± 1.7, p > 0.05 for all comparisons).

### Post-operative findings

#### Primary outcomes

A negative correlation was found between post-operative PNIF and RMR (r = – 0.336, p = 0.005). This association remained robust even after adjusting for potential confounders such as age, sex, and height in a linear regression model (β = – 0.043, p = 0.017), suggesting that post-operative PNIF may be an independent predictor of RMR (see Fig. 2).

Post-operative RMR was significantly higher in the SRP group (33.9 ± 5.8 kcal/Kg/day) compared to the ST group (29.1 ± 6.4 kcal/Kg/day, p = 0.005). This significance persisted after adjusting for age, sex and height (β = 4.530, p = 0.023). Nevertheless, when post-operative PNIF is inserted as a factor, the association between SRP and higher pre-operative RMR was no longer significant (β = 3.536, p = 0.085). In fact, STR patients showed lower post-operative PNIF values compared to ST (STR: 93.8 ± 33.1 vs ST: 123.9 ± 44.1, p = 0.005).

Likewise, post-operative RMR was significantly higher in SRP (33.9 ± 5.8 kcal/Kg/day) compared to IRT (22.1 ± 4.4 kcal/Kg/day, p = 0.004). This significance persisted after adjusting for age, sex, height and post-operative PNIF (β = 4.057, p = 0.026).

#### Secondary outcomes

No differences were found in any measurement of body composition (p > 0.05 for between groups) and baseline subjective measurements between groups (SNOT-22 – IRT: 8 ± 3; ST: 22.9 ± 16.5, SRP: 20 ± 15.5, p > 0.05 for all comparisons; Epworth – IRT: 4 ± 2 vs ST: 6.8 ± 4.1, SRT: 8 ± 6.6, p > 0.05 for all comparisons; SF-36 – IRT: 28 ± 5; ST: 28.1 ± 3.2, SRP: 29 ± 1, p > 0.05 for all comparisons).

### Pre to post-operative changes

#### Primary outcomes

PNIF showed significant improvement across all surgical groups (Table 2).

No statistically significant changes were observed in RMR across any group after surgical intervention (see Table 2).

To investigate whether the observed improvement in PNIF post-surgery was associated with changes in RMR, change scores (Δ) were calculated for both PNIF (ΔPNIF) and RMR (ΔRMR). However, no statistically significant association was found between ΔPNIF and ΔRMR (p = 0.536).

#### Secondary outcomes

All the subjective measurements (SNOT-22, Epworth and and SF-36) improved after surgery (see Table 3). Importantly, there were also some significant variations in body composition with surgery, especially regarding total body weight, muscle mass, and water composition (see Table 4).

**Table 3:** Comparative analysis of pre- and post-operative values of subjective measurements

	Pre-op mean (± SD)	Post-op mean (± SD)	P-value
Patient-Reported Outcome measures (PROMs)			
SNOT-22	43.9 ± 19.3	22.4 ± 16.3	< 0.001
Epworth	9.6 ± 5.1	6.8 ± 4.3	0.002
SF-36	25.3 ± 4	28.1 ± 3.3	< 0.001

*SD* standard deviation

Results in the table describe the analysis by using paired samples T-test

**Table 4:** Comparative analysis of pre- and post-operative values of body composition

Parameter	Pre-op mean (± SD)	Post-op mean (± SD)	P-value
Total Weight (Kg)	74 ± 14.6	75.6 ± 15.5	< 0.001
BMI	24.6 ± 3.7	25.1 ± 3.9	< 0.001
Body fat (%)	27.1 ± 8	26.5 ± 10.7	0.733
Muscle mass (Kg)	52.3 ± 12	55.5 ± 14	< 0.001
Total body water (%)	50.2 ± 7.2	53.9 ± 8.7	0.004
Visceral fat level	6.8 ± 3.9	6.5 ± 4	0.171
RMR (Harris-Benedict) (Kcal/day)	1643.2 ± 366.2	1684.5 ± 429.5	0.359

*SD* standard deviation, *BMI* body mass index, *RMR* resting metabolic rate

Results in the table describe the analysis by using paired samples T-test

## Discussion

NB may exert a more significant influence on human physiology than previously recognized. While its role in optimizing respiration and cardiovascular function is well-established [1–8], the potential influence of NB on metabolic function remains largely unexplored. To address this knowledge gap, this study investigated the relationship between NB and RMR, while assessing the impact of nasal surgery on RMR.

### Key findings and interpretation

**Higher RMR in SRP Group:** Patients undergoing SRP exhibited significantly higher post-operative RMR than those undergoing ST alone. This difference persisted after adjusting for potential confounders such as age, sex, and height. Nevertheless, the association between SRP and RMR disappeared when PNIF was included in the analysis. This key finding indicates that the difference in RMR between the groups was influenced by nasal airflow, with SRP patients showing lower PNIF values post-operatively compared to other groups. This observation may translate nasal airflow interference in RMR, with lower PNIF relating to higher RMR.

**PNIF as a Predictor of RMR:** Pre-operative and post-operative PNIF values were significantly correlated with RMR. This correlation was negative, meaning higher PNIF predicted lower RMR values and vice-versa. After confounder adjustment methods, post-operative PNIF emerged as an independent predictor of post-operative RMR. This observation aligns with earlier research by Singhal and Vishwakarma (1987), who highlighted the role of NB in energy expenditure, demonstrating that individuals with nasal obstruction exhibited increased metabolic activity compared to those with normal NB [9].

**Pre to post-operative changes in RMR and PNIF:** While surgical intervention significantly improved PNIF across all groups, a corresponding change in RMR was not observed. Changes in PNIF (ΔPNIF) did not correlate with RMR (ΔRMR) changes. This dissociation suggests that the relationship between nasal airflow and metabolic function is more nuanced than initially anticipated. It is plausible that the impact of surgical modifications on RMR extends beyond simple alterations in nasal airway resistance. Factors such as changes in nasal geometry, mucosal function, or even autonomic nervous system activity may mediate the metabolic response to surgical intervention.

**Increase in muscle mass:** surprisingly, there was a statistically significant increase in muscle mass after surgery. This finding may relate to a potential anabolic effect of improved nasal breathing. Enhanced oxygen delivery and utilization due to improved nasal breathing could contribute to muscle protein synthesis. Studies have shown that hypoxia can impair muscle protein synthesis and promote muscle atrophy [21, 22]. Conversely, improved oxygenation may create a more favorable environment for muscle growth. On the other hand, nasal surgery can improve olfactory and gustatory perception by increasing airflow and reducing nasal congestion [23, 24]. This enhanced sensory experience may lead to increased enjoyment of food, potentially promoting greater food intake and contributing to the observed increase in muscle mass [25, 26]. Nevertheless, it is essential to consider the potential influence of peri-operative factors, such as fluid shifts, inflammation, and medication use after surgery, on body composition changes.

### Metabolic response to other surgical procedures

It is worth noting that studies investigating other surgical procedures have also reported that RMR remains stable after surgery. For instance, research on patients undergoing cardiac surgery with moderate hypothermic cardiopulmonary bypass has shown that no significant alteration in RMR was observed on the first 6 postoperative days [27]. Similarly, patients who underwent major abdominal surgery showed stable RMR values in postoperative days 3 and 5 [28]. This aligns with the findings of the present study, which demonstrated no significant RMR differences 3 months after nasal surgery, although favoring a correlation between RMR and PNIF at each time point.

While this study observed an anabolic effect with increased weight and muscle mass after nasal surgery, research on other head and neck interventions, such as orthognathic procedures, has shown a contrasting trend towards weight loss [29]. This discrepancy may be attributed to post-operative pain interfering with feeding, highlighting the diverse metabolic responses across different surgical procedures.

It is important to acknowledge that the majority of research on surgical effects on RMR focuses on bariatric surgery, where the primary goal is to induce metabolic alterations [30]. Therefore, findings from bariatric surgery studies may not be directly comparable to the metabolic responses observed in other surgical specialties, including nasal surgery.

### Potential mechanisms of association: nasal breathing and RMR

Several mechanisms could explain the observed association between nasal breathing and RMR.

**Respiratory effort**: It is plausible that alterations in respiratory mechanics and energy expenditure mediate the observed association between PNIF and RMR. Increased nasal airway resistance can lead to higher intrathoracic pressure during inspiration, necessitating greater activation and muscular tone of the diaphragm and intercostal muscles to achieve adequate ventilation [31]. This heightened respiratory effort increases energy expenditure, which could contribute to a higher RMR [32, 33].

**Autonomic Nervous System Activity:** Sensory receptors in the nasal cavity can detect changes in airflow and temperature, relaying this information to the brainstem and influencing autonomic outflow [34, 35]. NB can stimulate parasympathetic branches of the autonomic nervous system, depending on airflow patterns [9]. Increased parasympathetic activity may decrease heart rate and blood pressure [25]. Therefore, NB patterns favoring parasympathetic activation could reduce RMR and promote an anabolic response.

**Hormonal Regulation:** Nasal breathing might modulate the production and release of hormones involved in energy balance, such as leptin [36]. Primarily produced by adipose tissue, leptin acts as a satiety signal, reducing appetite and increasing energy expenditure [36, 37]. Studies described lowering of leptin levels in patients with upper airway obstruction after treatment [38, 39]. This raises the possibility that improved NB after surgery could impact circulating leptin levels. In that case, increased appetite and reduced energy expenditure could contribute to observed significant weight gain.

### Implications for metabolic health

The potential impact of NB on RMR has significant implications for metabolic health. Given the rising prevalence of obesity and metabolic disorders worldwide, understanding the factors influencing energy expenditure and metabolic regulation is crucial.

If NB can relate to RMR, optimizing nasal airflow through medical or surgical interventions may benefit individuals with upper airway-related breathing disorders.

### Limitations and future directions

This study has several limitations. The sample size was relatively small, potentially limiting the generalizability of the findings. Furthermore, the absence of a control group makes it difficult to take illations about the observed changes with surgery. The inclusion of a control group in future studies, such as patients undergoing unrelated surgical procedures or individuals with nasal obstruction who are not undergoing surgery, would help to isolate the specific effects of nasal surgery on metabolic function. The short follow-up period also restricts the ability to assess long-term trends and the sustainability of any observed effects. It is possible that the observed changes in weight and muscle mass may be influenced by peri-operative factors and may not persist in the long term. Future research with longer follow-up periods (e.g., 1 year after treatment) would be valuable to assess the durability of these changes. Furthermore, it is important to consider the potential iatrogenic effects of post-operative treatment on metabolic function. Medications such as analgesics and anti-inflammatories could potentially influence metabolic parameters and body composition. To mitigate the potential confounding effects of general anesthesia and allow healing, post-operative measurements were conducted at 3 months after surgery. This decision was based on research indicating that RMR typically returns to preoperative levels shortly after surgery (usually within 24 h) [40]. Regarding the impact of surgery on muscle mass, there is limited objective data on the direct effects of anesthesia. Existing studies primarily focus on critically ill patients who experience prolonged periods of sedation and muscle relaxants in the intensive care setting [41]. These circumstances differ significantly from the relatively short duration of anesthesia and post-operative recovery in this study´s population. Finally, this work did not fully account for confounding factors such as diet, exercise, medications, stress, and sleep, which could independently influence RMR measurements and body composition. Future research should address these limitations by including larger populations, incorporating a control group, extending the follow-up period, and further controlling for potential confounding factors.

## Conclusion

While preliminary, these findings suggest that NB may be a factor in energy homeostasis. Interventions to optimize nasal airflow could offer benefits beyond addressing nasal obstruction. However, further research is needed to fully elucidate the underlying mechanisms, explore long-term effects, and evaluate the effectiveness of interventions in diverse populations and clinical applications.

## Data Availability

There are no publicly available datasets related to this work.

## Declarations

### Data confidentiality

The authors declare that they followed the protocols of their work in publishing patient data.

### Human and animal protection

The authors declare that the procedures followed are in accordance with the regulations established by the directors of the Commission for Clinical Research and Ethics and in accordance with the Declaration of Helsinki of the World Medical Association. Approval was granted by the Ethics Committee of CHUdSA/ICBAS (2021–234(186-DEFI/194-CE)).
